# Animal Chemistry, or Organic Chemistry in Its Applications to Physiology and Pathology

**Published:** 1842-09-24

**Authors:** M. Clymer


### BIBLIOGRAPHICAL NOTICES.

*Animal Chemistry, or Organic Chemistry in its applications to Physiology and Pathology.* By Justus Liebig, M. D., &c. Professor of Chemistry in the University of Giessen, Edited from the author's manuscript by William Gregory, M. D., F. R. S. E., M. R. I. A. &c. New York: Wiley & Putnam, 1842. 8vo. pp. 856.

We had an opportunity of anticipating in our 30th number, the publication of a considerable portion of this work, and gave a pretty full analysis of Professor Liebig's views of vital action in animals, and of his theory of animal heat, and of the cause of death in chronic disease. The present work is one of transcendant ability, and is evidently the result of years of patient and acute investigation, of deep and original thought, and of the most intimate acquaintance with the sciences the author has called to his aid in the arduous pursuit he has undertaken. If we cannot in every instance yield our cordial assent to the premises, yet, granting these, we are forced to admire the beautifully sustained chain of reasoning, pervading every page, and stated with all the clearness and precision of a mathematical demonstration. The work is eminently suggestive, and the paths which the genius of Dr. Liebig has indicated, will doubtless open up others, which resolving the difficult problems of vitality, will create a new physiology and a rational pathology.

We recognize in the ovum as well as in the seed of the plant, a certain force in a state of rest, which is the source of growth and of reproduction. The static equilibrium of this force is disturbed by impregnation, and by the presence of air and moisture, and its new dynamic condition is manifested by the production of a series of forms. The increase of the organic mass is determined by the passage of matter from a state of rest to one of motion—assimilation, or the process of formation and growth. By this means certain component parts of the nourishment taken become component parts of the mass, and by comparing its chemical constitution with that of the nourishment, we ascertain with certainty what component parts of the latter have been assimilated and what rejected. The animal organism demands for its support, in all circumstances, highly organized atoms. In vegetables the whole process of formation is due to external influences, but in the animal organism, developement is, to a great degree, independent of these influences, it possessing in itself the source of motion. Every atom in the animal economy is produced from a peculiar fluid, circulating through every portion of it. Ordinary experience informs us that there is a perpetual change of matter going on in the organism, a constant transformation of organized into unorganized structure, which losing its condition of vitality, must be renewed. Every manifestation of force, every motion, every thought, every sensation is followed by a change of structure. All vital activity arises from the mutual action of the elements of the food and the oxygen of the air, and this is the source of the animal heat.* The only known ultimate cause of vital force is a chemical process. If this be impeded or fail, the manifestation of the phenomena of life ceases. In the animal organism motion invariably proceeds from the nervous apparatus ; we call, therefore, the production of force, the dynamic phenomena in the animal body, *nervous life*, and the resistance, the condition of static equilibrium, *vegetative life*. The passage of matter from a state of motion to a state of rest appears in an increase of the mass, and in the supply of waste ; while the motion itself, or the production of force, appears in the shape of waste of matter. Now in the young animal the waste is less than the increase, and this condition does not cease in the female with complete developement, as in the male; and thus in her, vegetative life is more intense than nervous life. If nutrition is dependant on the blood, then those substances only can be regarded as food which are susceptible of conversion into blood.

To determine, therefore, what substances are nutritious, we must ascertain the composition of the food, and compare it with that of the ingredients of the blood. When blood is withdrawn from the circulation, it is well known that it separates into a yellowsih liquid, called the *serum* of the blood, and a gelatinous mass, which, when coagulating blood is briskly stirred, adheres to the rod. This is the *fibrine* of the blood, which is identical with muscular fibre, when this is purified from all foreign matters. The second principal ingredient resides in the serum, and possesses all the properties of white of eggs, and when heated coagulates into a white elastic mass, and is known as *albumen*. Chemical analysis has led to the remarkable result that fibrine and albumen contain the same organic elements, united in the same proportion. Their particles are arranged in a different order, as is shown by the difference of their external properties, but in their chemical composition, the ultimate arrangement of their organic elements, they are identical, and Denis has recently actually succeeded in converting fibrine into albumen. In the process of nutrition both albumen and fibrine are capable of conversion into muscular fibre, and muscular fibre into blood. No part of an organic mass which possesses life and motion is destitute of nitrogen; all of them also contain carbon and the elements of water, but the latter in no case in the proportion to form water. The chief ingredients of the blood contain nearly 17 per cent. of nitrogen. The animal body is incapable of producing an elementary body out of substances which do not contain it; it is obvious then that all kinds of food must contain a certain proportion of nitrogen, for this element is essential to the composition of the animal body, which is incapable of creating it, and no nitrogen is absorbed from the air in the vital process.

* For the developement of these views, see No. 30 of this volume, p. 465.

The simplest example of the nutritive process is seen in the carnivora, who live on the graminivora, whose flesh and blood are identical with their own. The food of graminivorous animals consists of vegetables, a large portion of which contain but little nitrogen. From what substances, then, is the blood, from whence their organs are developed, derived? Chemical research shows us that all the parts of vegetables, susceptible of affording nutriment to animals, are rich in nitrogen. These nitrogenized compounds are *vegetable albumen*, *vegetable fibrine*, and *vegetable caseine*, and are the true constituents of the food of graminivorous animals, and which contribute to the increase of the mass in their body. These three substances, contain the same organic elements, united in the same proportion by weight, and are moreover, identical in composition with the chief constituents of the blood—animal fibrine and albumen. By these discoveries how simple is the process of nutrition in animals. The vegetable principles, which in animals form the blood, contain its chief constituents ready formed, so far as regards composition. A certain quantity of iron, besides, exists in all plants, and this appears in the blood discs. By these principles the graminivora obtain in their food the identical principles, on the presence of which the nutrition of the carnivora depends.

Having thus seen the mode of growth in animals, an important problem remains to be solved—What is the function of those substances which contain no nitrogen, as starch, sugar, gum, pectine, &c.? The graminivora cannot live without these substances; and the nutrition of the young of carnivora is accomplished by similar means, for their developement is dependant on the supply of milk, which contains but one *nitrogenized* ingredient, *caseine*. But milk contains, besides, butter (fat) and sugar of milk, which are substances rich in carbon and the elements of water, and are indispensable to life. By means of these constituents the organism is protected from the action of the oxygen of the atmosphere; for the nitrogenized elements of the food contain exactly the amount of carbon requisite for the formation of fibrine and albumen, and this excess is expended in the production of animal heat. The bile, too, plays an important part in the elaboration of the carbon of the food, and on its peculiar use in the animal economy Dr. Liebig has constructed a very pretty theory. When graminivora and carnivora are deprived of exercise they consume more food than is requisite to reproduce the waste, and at the same time more carbon and hydrogen are introduced into the system than is necessary to support respiration and maintain the animal heat. Now want of exercise and diminished cooling are equivalent to a deficient supply of oxygen. There is, therefore, an excess of carbon and this manifests itself in the production of *fat*. This is an anormal condition, and depends on the disproportion of carbon in the food and of the oxygen absorbed by the lungs and skin. This excess of carbon is never seen in wild animals, nor in the Arab of the Desert; but prevails among the inmates of prisons, and the sedentary females of eastern climes, and in stall-fed animals. Its formation depends on a deficiency of oxygen. When animals are fattened on food destitute of nitrogen, only certain parts of its structure increase in size. Compare the liver of an ordinarily fed goose, which is firm and elastic, to the soft, spongy organ of the imprisoned animal. The essential difference is in the greater or less deposition of fat in the cells, causing their expansion. In some diseases the starch, sugar, &c., of the food do not undergo the changes which enable them to assist in respiration, and consequently to be converted into fat. Thus in diabetes mellitus the starch is only converted into grape sugar, and is expelled from the body without further change. In inflammation of the liver the blood is loaded with fat and oil, and it is probable that some of the constituents of the bile are converted into fat. The nutritious elements of the food may be divided into two classes: the first is nitrogenized, and is susceptible of conversion into blood, and contains what may be called the *plastic elements of nutrition;* they are—vegetable fibrine, vegetable albumen, vegetable caseine, animal flesh, animal blood. The second class is *now nitrogenized* and is incapable of this transformation; it serves to support the process of respiration, and its contents may be called the elements of respiration; they are—fat, starch, gum, cane sugar, grape sugar, sugar of milk, pectine, barsorine, wine, beer, and spirits.

The second part of Professor Liebig's work is devoted to the consideration of the metamorphosis of the tissues. When animal albumen, fibrine and caseine, are dissolved in a moderately strong solution of caustic potash, and then exposed to a high temperature, decomposition occurs, and on the addition of acetic acid, a translucent, gelatinous precipitate occurs, of the same character and composition, from which ever of the thin substances it has been produced. This compound may be considered as the starting point of all the other animal tissues, and Mulder gave to it the name of *proteine*, (from πρωτευω, I take the first rank.) The blood is consequently a compound of proteine, with inorganic substances in variable proportions. All the organic nitrogenized constituents of the body, however different may be their composition, are derived from proteine. The development of the young animal from the egg of the fowl proves this.

A new office is given to the saliva by our author—the direct introduction of oxygen into the stomach. During mastication there is an increased secretion of saliva, and this possesses the property of enclosing air in the shape of froth in a greater degree than even soapsuds. In this manner the air reaches the stomach, when its oxygen enters into combination, and its nitrogen is exhaled through the skin and lungs. The longer the continuance of digestion, the more copious the secretion, and the larger the quantity of air which enters the stomach. The gelatinous tissue is not a compound of proteine; and animals fed exclusively with gelatine die, because the gelatinous tissues are incapable of conversion into blood. When mixed with other food it nourishes the gelatinous tissues, and thus diminishes materially the productive energy.

The supposed function of the bile is highly ingenious. It neither assists digestion, nor is it an excretion. It is the fuel which supplies the respiratory process, and assists in the production of animal heat.

The identity of *theine* and *caffeine*—the active principles of tea and coffee—has been recently established by chemistry. The action must consequently be similar on the system. Now they are both highly nitrogenized, and one ingredient of the bile, *taurine*, contains also a great deal of nitrogen; and Dr. Liebig believes that theine and caffeine are active in the production of this constituent. When there is a consumption of an excess of non-azotized food, or there is but little movement, then the use of tea and coffee may be beneficial, for a nitrogenized product is thus furnished. Tea would be injurious to the Indian, for the waste in him is already great enough, and to retard this transformation of matter he resorts to tobacco, and assists its action with brandy. Tobacco being antiseptic, it retards the digestive process; hence cigar smoking after dinner must impede digestion, and be generally injurious. Dr. Liebig's theory of the mode of action of the vegetable alkaloids is highly ingenious and plausible. The substance of the brain and nerves is produced from the elements of vegetable albumen, fibrine and caseine, either alone, or with the aid of non-azotized food, or the fat formed from it; and as the active principles of bark and opium may be converted into the constituents of the nervous matter, the most probable supposition of their action is, that they assist in forming new, or in transforming existing nervous matter.

The third and last part is devoted to the consideration of the phenomena of motion in the animal organism, and though eminently speculative, contains much which is highly interesting to the physician.

M. Clymer.

